# Supplementary material for: Intermediate-state-trapped mutants pinpoint G protein-coupled receptor conformational allostery
Source: Nat Commun. 2023 Mar 10;14:1325. doi: 10.1038/s41467-023-36971-6 (PMC10006191; doi:10.1038/s41467-023-36971-6)

Supplementary Information

Intermediate-state-trapped Mutants Pinpoint G Protein-coupled Receptor Conformational Allostery

Xudong Wang[1†], Chris Neale[2†], Soo-Kyung Kim[3], William A. Goddard[3], Libin Ye[1,4*]

[1]Department of Cell Biology, Microbiology and Molecular Biology, University of South Florida, Tampa, FL, USA 33620

[2]Theoretical Biology and Biophysics, Los Alamos National Laboratory, Los Alamos, NM, USA 87545

[3]Materials and Process Simulation Center (139-74), California Institute of Technology, Pasadena, CA, USA 91125

[4]H. Lee Moffitt Cancer Center & Research Institute, 12902 USF Magnolia Drive, Tampa, FL, USA 33612

[†]These authors contributed equally to this work.

***Corresponding author**

Libin Ye, Assistant Professor

Department of Cell Biology, Microbiology, and Molecular Biology, University of South Florida, Tampa, FL, USA 33620; H. Lee Moffitt Cancer Center & Research Institute, 12902 USF Magnolia Drive, Tampa, FL, USA 33612

E-mail: libinye@usf.edu Tel: 1-813-974-6007

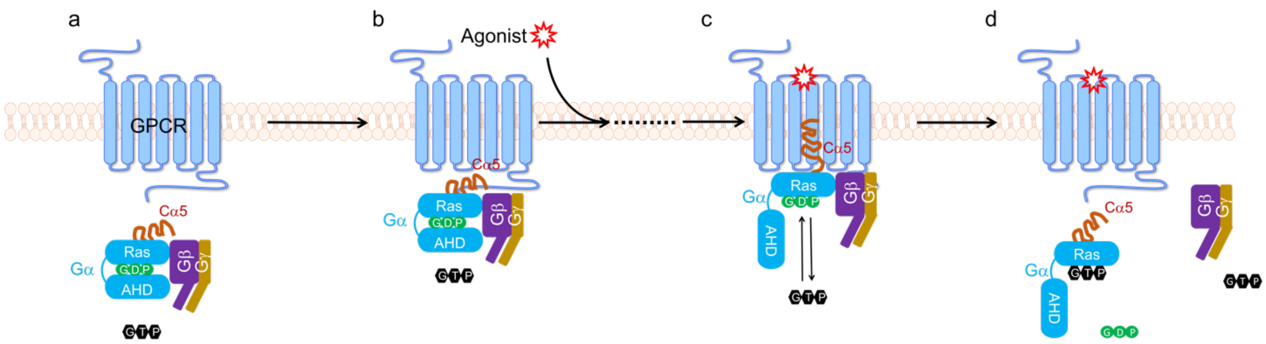

Supplementary Figure 1: A typical GPCR activation process based on static structures. a Inactive state of the receptor and Gαβγ. **b** Pre-coupled GPCR-Gαβγ. **c** GDP bound Gαβγ engages the receptor, triggering the displacement of the Cα5 helix and GDP release. **d** Upon activation, Gαβγ dissociates.

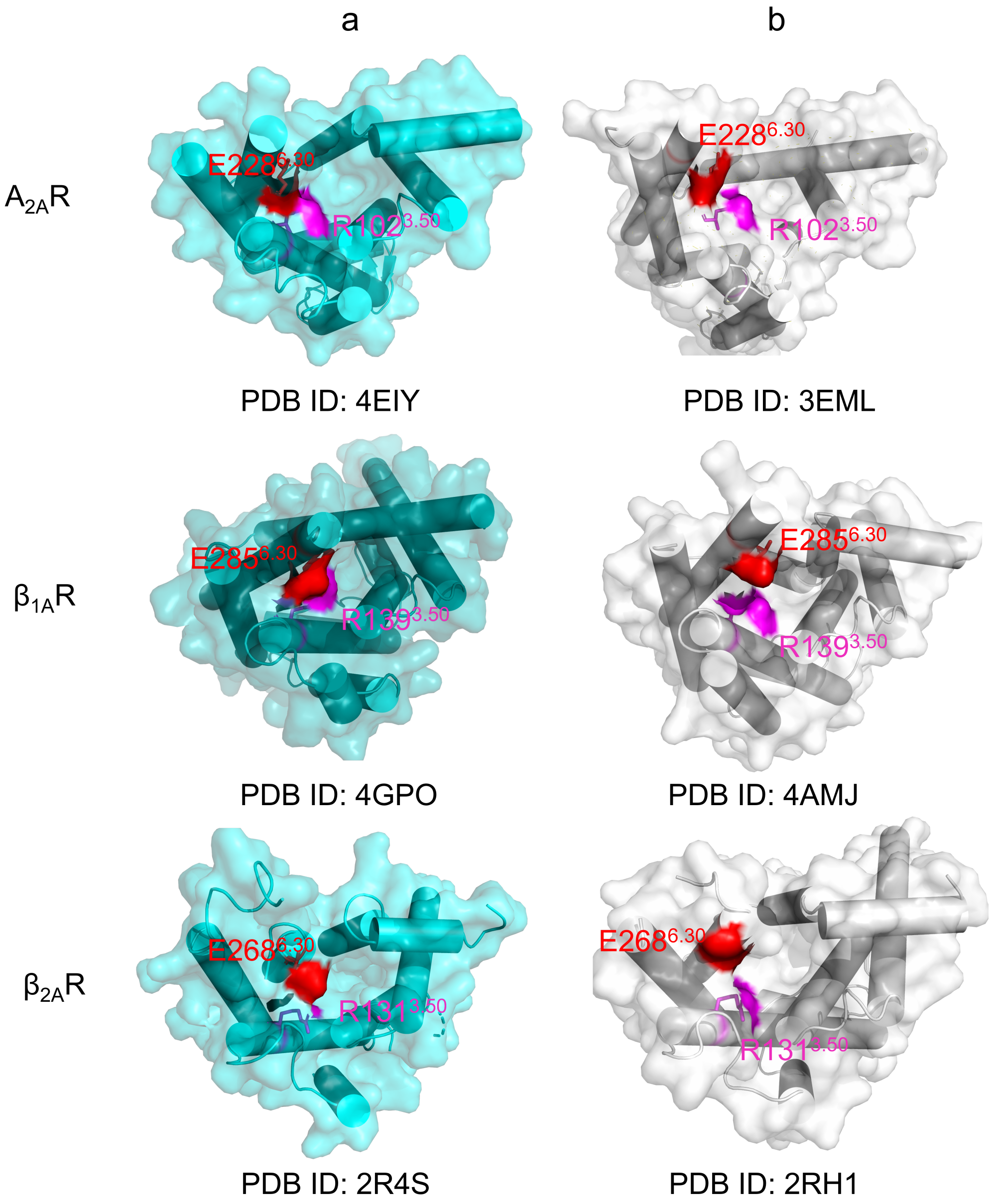

Supplementary Figure 2: Ionic lock (DR$^{3.50}$Y-E$^{6.30}$) between TM3 and TM6 in two different inactive conformational structures for each of three GPCRs. **a** Ionic lock ON structures for different resolved GPCRs (cyan). **b** Ionic lock OFF structures for different resolved GPCRs (gray).

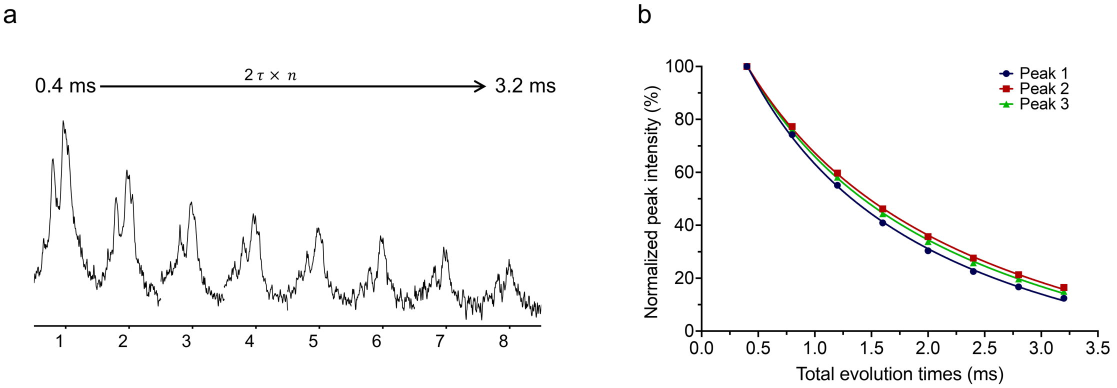

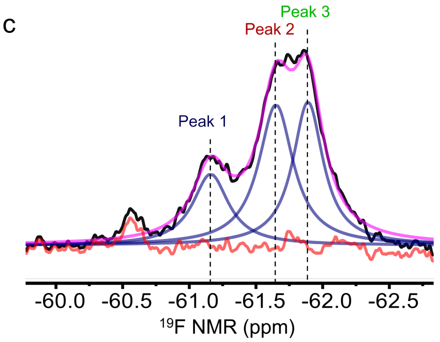

d

	$\Delta\upsilon_{1/2}$ from T_2 measurement (Hz)	$\Delta\upsilon_{1/2}$ 3-state deconvolution (Hz)
Peak 1	237.2 ± 9.7	183.5
Peak 2	205.0 ±11.3	304.7
Peak 3	215.5 ±13.1	155.5

Supplementary Figure 3: Linewidth determination for different conformational states. **a** T2 relaxation experiments for linewidth measurements of different resonances. **b** T2 relaxation fitting and the corresponding linewidth from the fitting shown in (**d**). **c** 2-state based spectral deconvolution for the major resonance that consists of Peak 2 and Peak3. **d** The linewidths of each deconvoluted resonance, suggesting the major resonance contains at least three conformational components instead of two, which led us to propose the 5-state activation model in this manuscript, consistent with the observations from conformation-biased mutants.

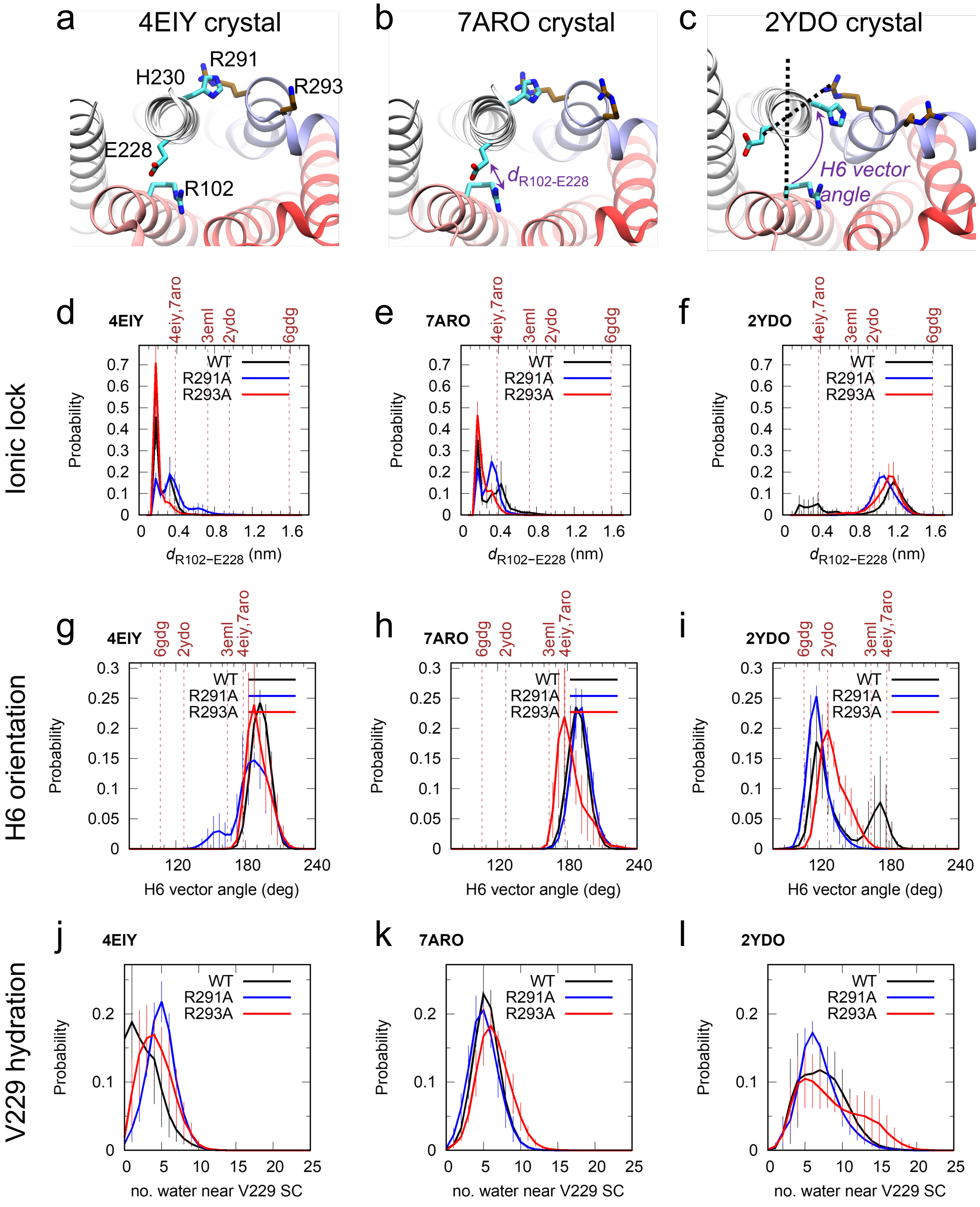

Supplementary Figure 4: Crystal structures and simulation behavior along the activation profile. a-c Crystal structures of the $A_{2A}R$ obtained in the presence of **a** inverse agonist ZM241385 (PDB ID: 4EIY), **b** partial agonist LUF5833 (PDB ID: 7ARO), or **c** full agonist adenosine (PDB ID:

2YDO). Protein colored red to blue from N- to C-terminus. **d-f** Ionic lock displacements, $d_{R102\text{-}E228}$, sampled in three 1 μs repeat simulations for each of the WT, R291A and R293A systems, starting from PDB IDs **d** 4EIY, **e** 7ARO, and **f** 2YDO. Vertical bars represent the standard error of the mean from three independent simulations. Distances observed in 5 crystal structures are denoted by vertical dashed lines with PDB IDs indicated above the plots. Note that values of $d_{R102\text{-}E228}$ were reduced by 0.1 nm for crystal structures to account for missing hydrogen atoms. **g-I** TM6 orientation is quantified based on the angle between a vector from E228$^{6.30}$ to H230$^{6.32}$ and a vector from R102 to TM6 (see Methods). **j-l** Number of water molecules within 0.6 nm of residue V229$^{6.31}$.

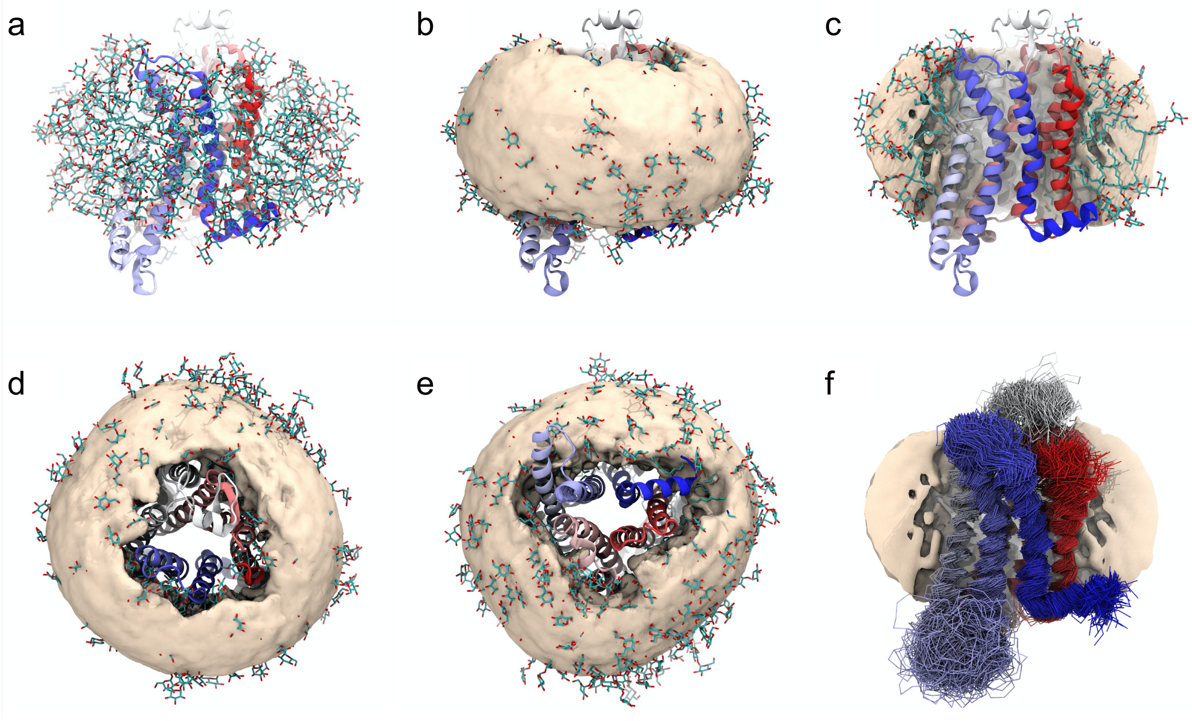

Supplementary Figure 5: Detergent coverage of $A_{2A}R$ in MD simulations. **a** Representative structure after 1 μs of simulation. Protein (ribbons) colored red to blue from N- to C-terminus. MNG-3 detergents shown as sticks with carbon (cyan) and oxygen atoms (red); hydrogen atoms, water, and salt are omitted for clarity. **b** Representative structure from part (**a**) overlaid with an MNG-3 density isosurface (orange) that encloses regions with time- and ensemble-average MNG-3 densities >33.45 non-hydrogen atoms per nm^3 (approximately the density of oxygen atoms in liquid water). Data taken from 0.1-1 μs in all 27 simulations after least square fitting C_α atoms of the protein core. **c** Slice through the MNG-3 density profile shown in part B. **d** Extracellular view. **e** Intracellular view. **f** Aligned protein conformations from all 27 simulations, sampled every 20 ns, overlaid on a sliced average MNG-3 density isosurface.

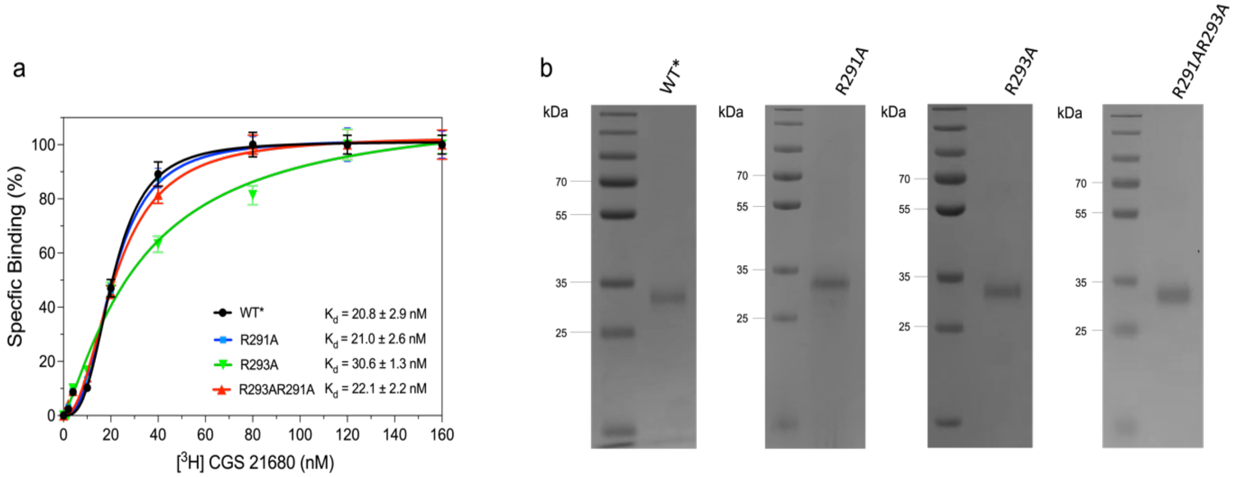

Supplementary Figure 6: Saturation binding assays and SDS-PAGE for purified WT* and conformation-biased mutants. a Saturation binding assays for the constructs used in this study; the source data for these measurements were included in the Source Data file. **b** SDS-PAGE for the constructs used in this study after XAC column purifications. The uncropped SDS-PAGE images were supplemented at the end of this document.

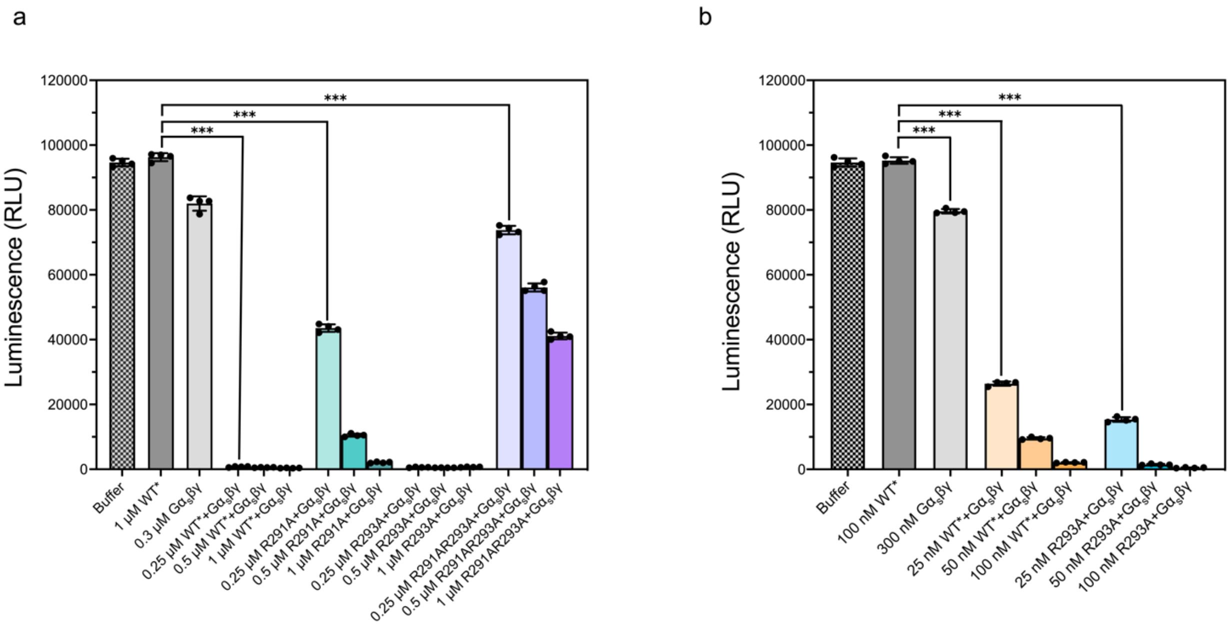

Supplementary Figure 7: GTP hydrolysis assays for different mutants. **a** GTP hydrolysis assays for WT* and mutants R291A (teal), R293A, and R291AR293A (violet) as a function of receptor concentrations from 0.25 μM to 1 μM. **b** GTP hydrolysis capacity comparison between the WT* (orange) and R293A (cyan) with a low receptor concentration set from 25 nM to 100 nM. Of note, the references were normalized in two sets of measurements. Data with error bars are presented as mean±SEM of four independent experiments. Statistical analyses were performed using the ordinary one-way ANOVA followed the two-sides sidak's post-hoc test in PRISM 9.3.1, ***$p < 0.001$, in comparison to the WT*. The source data for these measurements were included in the Source Data file.

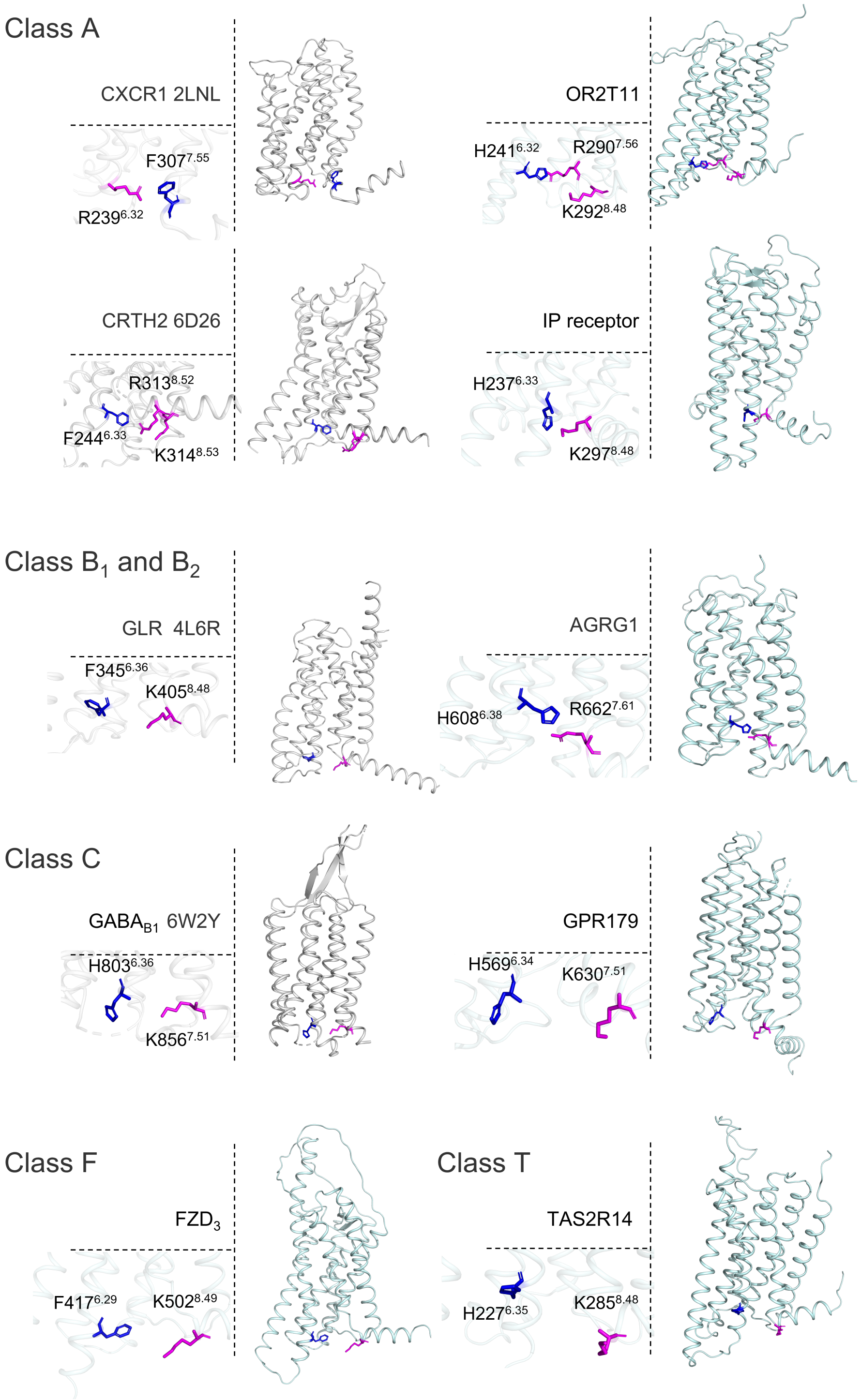

Supplementary Figure 8: Resolved (gray) and simulated (cerulean) GPCR structures from different families showing cation-π interactions between TM6 and TM7/H8.

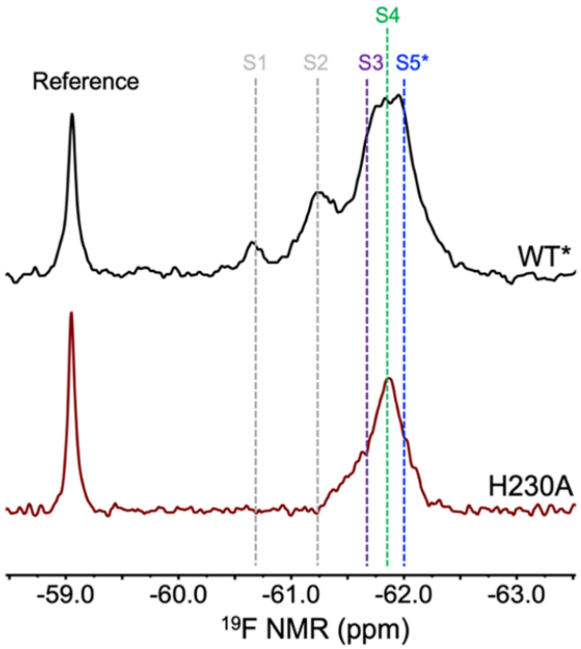

Supplementary Figure 9: ^{19}F-NMR spectrum of H230A (maroon) in comparison to the WT* (black).

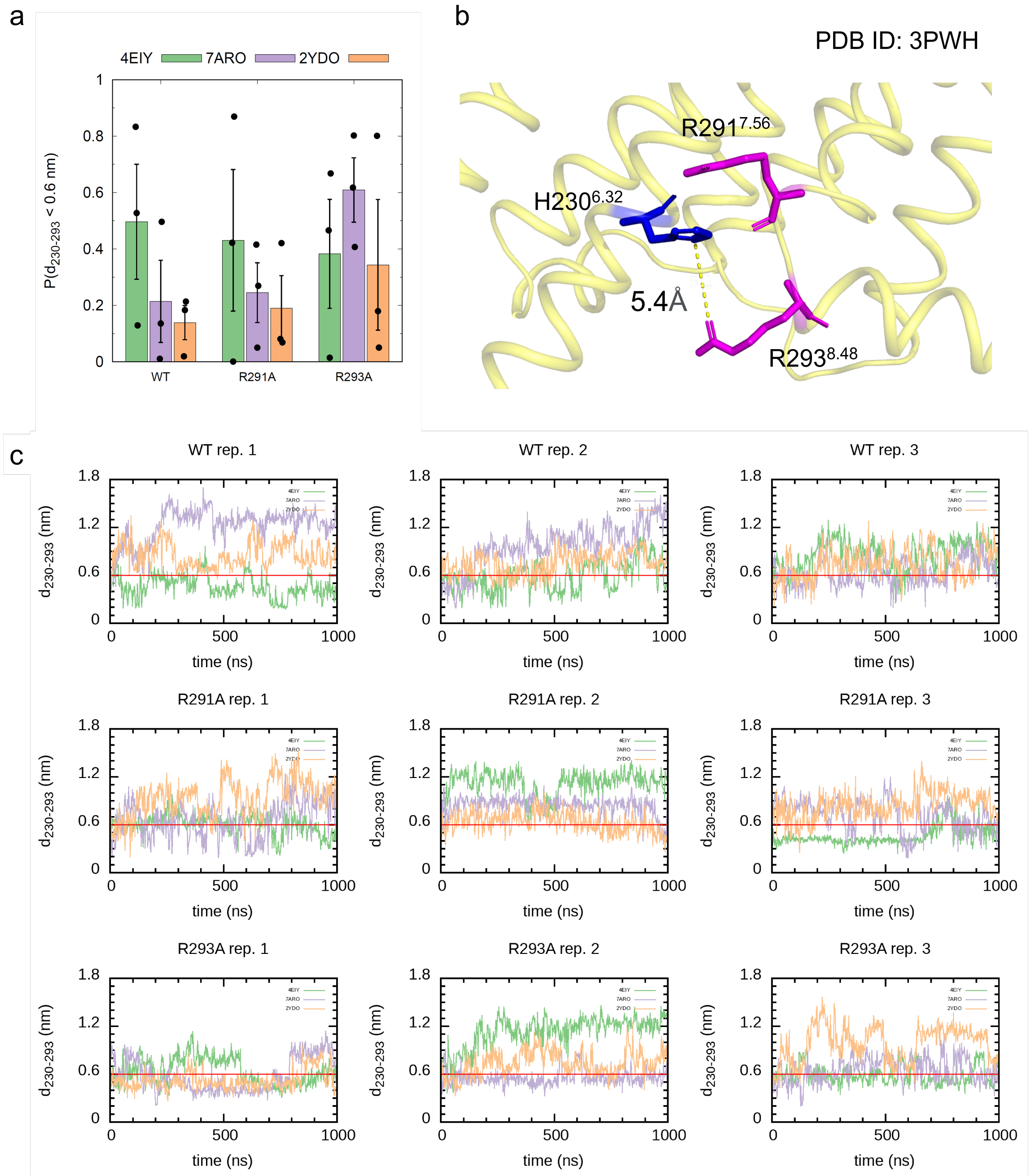

Supplementary Figure 10: H230$^{6.32}$-R293$^{8.48}$ interaction in MD simulation. a Probability that the minimum distance between H230$^{6.32}$ and residue 293$^{8.48}$, $d_{230\text{-}293}$, is less than 0.6 nm. Vertical bars represent the standard error of the average over three simulations. Data with error bars are presented as mean±SEM of three independent simulations. **b** Interaction

between H230$^{6.32}$ and R293$^{8.48}$ in the crystal structure PDB: 3PWH. **c** Time-series of $d_{230\text{-}293}$ in each of the 27 MD simulations, which started from PDB 4EIY (green), 7ARO (violet), or 2YDO (orange) and contained either WT receptor, the R291A mutant, or the R293A mutant, with three technical repeats for each combination.

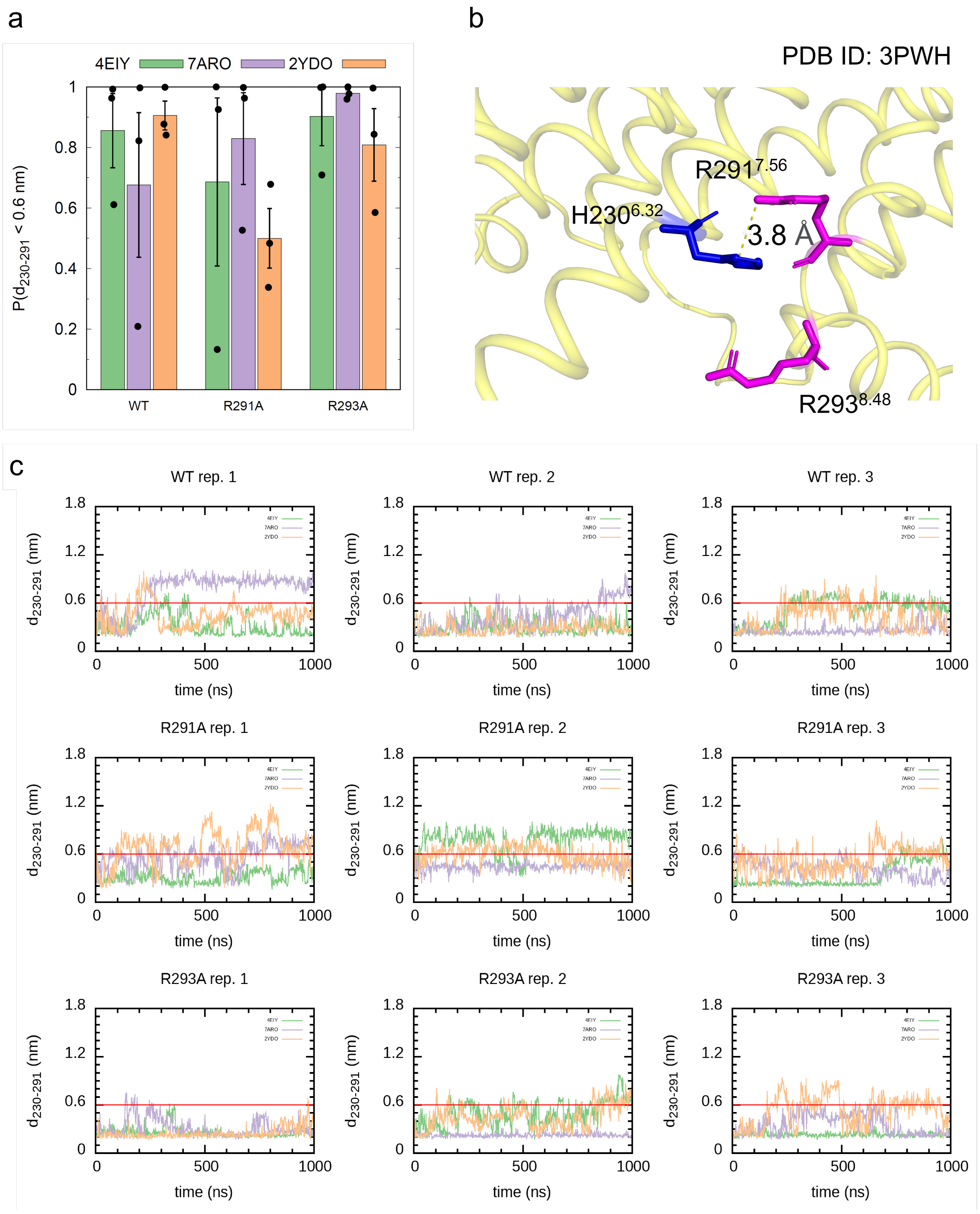

Supplementary Figure 11: H230$^{6.32}$-R291$^{7.56}$ interaction in MD simulation. a Probability that the minimum distance between H230$^{6.32}$ and residue 291$^{7.56}$, $d_{230\text{-}291}$, is less than 0.6 nm. Vertical bars represent the

standard error of the average over three simulations. Data with error bars are presented as mean±SEM of three independent simulations. **b** Interaction between H230$^{6.32}$ and R291$^{7.56}$ in the crystal structure PDB: 3PWH. **c** Time-series of $d_{230\text{-}291}$ in each of the 27 MD simulations, which started from PDB 4EIY (green), 7ARO (violet), or 2YDO (orange) and contained either WT receptor, the R291A mutant, or the R293A mutant, with three technical repeats for each combination.

Supplementary Table 1: Primers used in this study

Primer name	Primer sequence (5'-3')
A_{2A}R_R291A	CTT CAT TTA CGC CTA CGC TAT CCG CGA GTT CCG C
A_{2A}R_R293A	CGC CTA CCG TAT CGC CGA GTT CCG CCA G
A_{2A}R_R291AR293A	CCT TCA TTT ACG CCT ACG CTA TCG CCG AGT TCC GCC AGA CCT
A_{2A}R_H230A	CAG TGA CTT GGC AGC AGC GCA CTC CTT CTG CAG T
pPIC9K-F	GACTGGTTCCAATTGAC
pPIC9K-R	GGCAAATGGCATTCTGACA

Uncropped SDS-PAGE images for Supplementary Fig. 6b

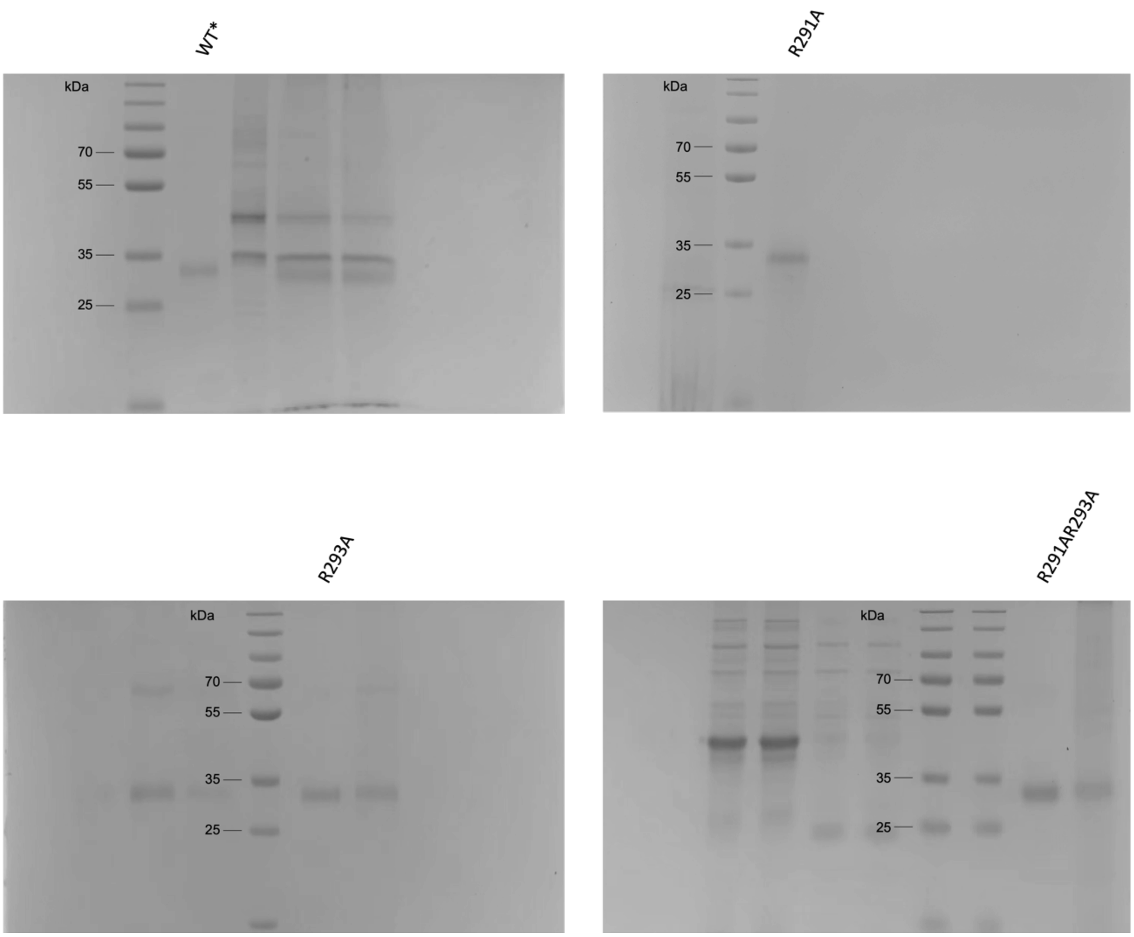

Supplement: Supplementary file 1 — Supplementary Information [file 41467_2023_36971_MOESM1_ESM.pdf]
